# Identification, mapping and relative quantitation of SARS-CoV-2 Spike glycopeptides by Mass-Retention Time Fingerprinting

**DOI:** 10.1038/s42003-021-02455-w

**Published:** 2021-08-03

**Authors:** Rod Chalk, William E. P. Greenland, Tiago Moreira, Jesse Coker, Shubhashish M. M. Mukhopadhyay, Eleanor Williams, Charlotte Manning, Tina Bohstedt, Rama McCrorie, Alejandra Fernandez-Cid, Nicola A. Burgess-Brown

**Affiliations:** 1 Centre for Medicines Discovery, ORCRB, University of Oxford, Oxford, UK; 2 Agilent Technologies, Lakeside, Cheadle Royal Business Park, Cheadle, Cheshire, UK

**Keywords:** Mass spectrometry, Glycobiology

## Abstract

We describe an analytical method for the identification, mapping and relative quantitation of glycopeptides from SARS-CoV-2 Spike protein. The method may be executed using a LC-TOF mass spectrometer, requires no specialized knowledge of glycan analysis and exploits the differential resolving power of reverse phase HPLC. While this separation technique resolves peptides with high efficiency, glycans are resolved poorly, if at all. Consequently, glycopeptides consisting of the same peptide bearing different glycan structures will all possess very similar retention times and co-elute. Rather than a disadvantage, we show that shared retention time can be used to map multiple glycan species to the same peptide and location. In combination with MSMS and pseudo MS3, we have constructed a detailed mass-retention time database for Spike glycopeptides. This database allows any accurate mass LC-MS laboratory to reliably identify and quantify Spike glycopeptides from a single overnight elastase digest in less than 90 minutes.

## Abstract (web summary)

Mapping multiple glycan species to the same peptide and location from shared retention time of glycopeptides with MS and HPLC, Chalk, Greenland et al. construct a detailed mass-retention time database for Spike protein glycopeptides. This allows any mass LC-MS laboratory to reliably identify and quantify Spike protein glycopeptides from a single overnight elastase protein digest in less than 90 minutes.

## Introduction

Glycosylation is known to play an important role in the efficacy and antigenicity of therapeutic proteins^1–3^. The current SARS-CoV-2 pandemic has spurred urgent research, much of it devoted to preparing vaccines, therapeutic antibodies, or antibody tests based on Spike protein, the virus’s primary surface antigen^4^. This 145-kDa protein forms a trimer^5^ with each subunit bearing twenty-two potential N-linked glycosylation sites and two O-linked sites of which approximately seventeen are occupied^5^. The unusually heavy and complex glycosylation observed in Spike protein is believed to play an important role in the pathogenicity of SARS-CoV-2 by mimicking host cell glycans and allowing the virus to evade the normal immune response^6^. Analysis of expressed Spike protein by mass spectrometry presents unique challenges in terms of its size and the number and complexity of its glycans. These challenges have been commendably met to date by laboratories with wide experience in glycan analysis and access to very sensitive, high-end nano-LC–MS–MS mass spectrometers^1,7–9^. However, in our laboratory and in others, a rapid and more robust methodology is needed for routine analysis of different batches of recombinant Spike protein. In addition, any method, which is reliant on LC–MS–MS of glycopeptides, may not necessarily detect specific glycans that fail to fragment under the conditions selected. LC–MS, by contrast, generates a mass, retention time, and relative abundance for all ionizable species. We have developed a simple Mass-Retention Time Fingerprinting (MRTF) method for mapping and relative quantitation of Spike glycopeptides. Overnight digestion using a single enzyme followed by a 65-min LC–MS run using any accurate mass instrument is the only experimental requirement. The resulting LC–MS data contain accurate mass, retention time and relative abundance values for each glycopeptide component. This dataset needs only to be matched against the preexisting Spike protein glycopeptide database reported here. We describe this method as “analytical mode”, which is both conceptually simple to understand, and straightforward to implement in any accurate mass LC–MS laboratory. For scientific completeness, we also describe the “discovery mode”, which we have used to generate the data for our Mass-Retention Time Fingerprinting database. However, there is no requirement for users to duplicate this discovery mode.

The database contains accurate mass and retention time signatures for 140 observed glycopeptides mapped at 13 sites, with a further 12 glycopeptides associated with six unassigned locations. By virtue of known mass and approximate retention time, a further 306-glycopeptide signatures were inferred. The database provided and the simplified analytical workflow is all that is necessary to characterize Spike protein glycans by MRTF.

## Results

**Fig. 1:**
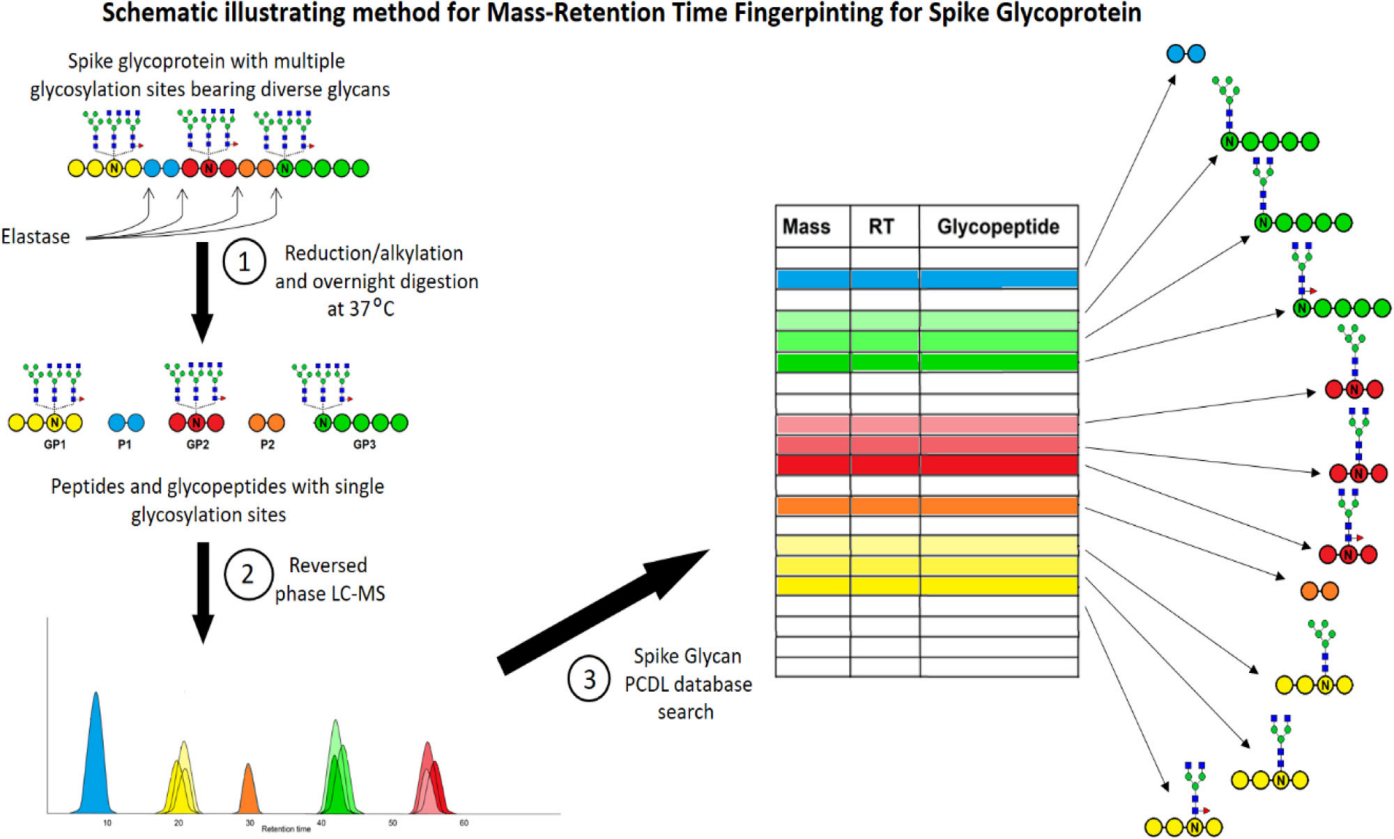
Schematic representation of the Mass-Retention Time Fingerprinting analytical workflow. Specific colors (yellow, blue, red, orange, and green) represent elastase-cleaved peptides. Shades of the same color represent three possible glycoforms of the same peptide, which resolve only partially by HPLC. The three workflow stages are **1** reduction/alkylation and elastase digestion. **2** Reversed phase LC–MS. **3** Database matching.

The simple MRTF workflow we describe as “analytical mode” is illustrated schematically in Fig. 1.

**Fig. 2:**
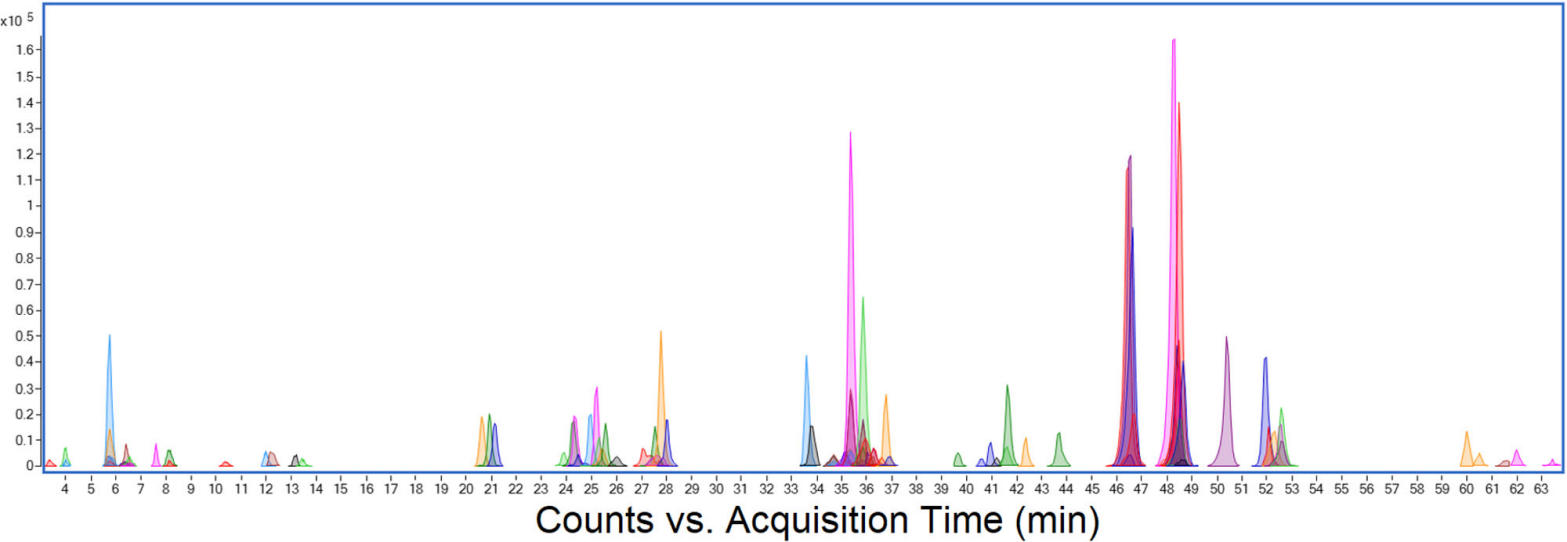
Combined extracted ion chromatogram (EIC) for 140 observed Spike protein glycopeptides. Combined extracted ion chromatogram (EIC) for 140 observed Spike protein glycopeptides separated by 60-min reverse-phase LC–MS run showing peak resolution for peptides of different sequence, and also different glycoforms of the same peptide, which are unresolved and coelute within a 4-min retention time window. Peak volume (area) can be used to estimate the relative abundance of each glycopeptide species.

**Fig. 3:**
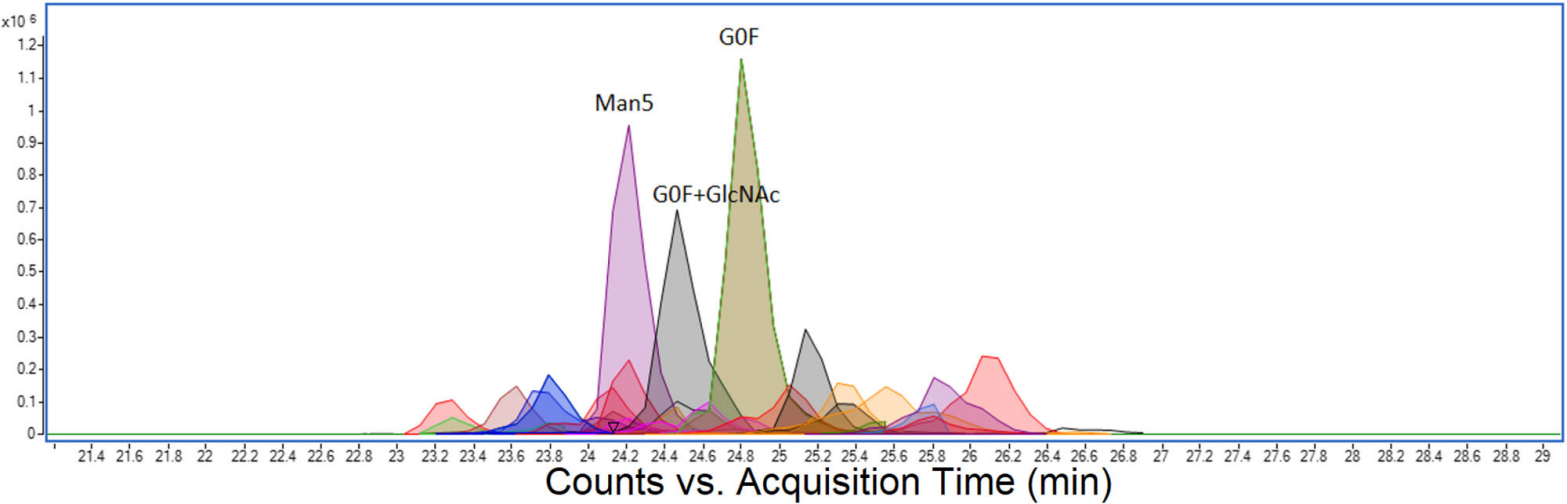
Combined extracted ion chromatogram (EIC) for 27 species of glycopeptide GEVFNAT (N343). All 27 glycoforms elute within +/− 2-min retention-time window. Only three glycans are labeled, the remainder are listed in the accompanying Table 1.

Combined extracted ion chromatograms are illustrated in Fig. 2. It can be seen that some species (peptides) are completely resolved, while many other species (glycans) coelute. Fine detail for the peak cluster around 24.5 min of retention time is shown in Fig. 3. This represents all observed glycoforms for the peptide sequence GEVFNAT at position N343, one of the two sites within the receptor-binding domain. Table 1 lists the retention times, masses, mass errors, and peak volumes (relative ion intensities) for these peaks. All 25 glycoforms have predictable retention times +/− 2 min, showing that retention time is almost entirely dictated by peptide sequence under these conditions.

**Table. 1:** Accurate masses and retention times for GEVFNAT glycopeptides.

Name	Mass	RT	Volume	ppm error
GEVFNAT Complex NeuAc (F)2	2838.1078	23.26	733674	−0.2
GEVFNAT Man8	2438.9113	23.30	744169	3.7
GEVFNAT Man7	2276.8615	23.61	1867525	2.7
GEVFNAT Complex NeuAc F	2692.0523	23.77	1266219	0.5
GEVFNAT Man6	2114.8091	23.80	2432950	2.7
GEVFNAT G1(F)2	2488.9768	24.09	1292274	2.8
GEVFNAT G2F	2504.9755	24.14	1007345	1.3
GEVFNAT Man4	1790.7051	24.20	3040952	2.3
GEVFNAT Man5	1952.7583	24.20	12492713	1.9
GEVFNAT Man3	1628.6504	24.20	666739	3.7
GEVFNAT G1F	2342.9183	24.37	1333546	3.3
GEVFNAT G0F	2180.8638	24.45	953034	4.3
GEVFNAT G0F+GlcNAc	2383.9436	24.47	9038631	3.7
GEVFNAT G1F	2342.9180	24.62	1090359	3.4
GEVFNAT G0	2034.8089	24.80	950868	3.1
GEVFNAT G0F	2180.8688	24.82	12980259	2.0
GEVFNAT A1(F)2-Gal+GlcNAc	2983.1427	24.91	732781	5.3
(G)EVFNAT G0	1977.7887	25.05	2947447	2.5
GEVFNAT G0F+GlcNAc	2383.9464	25.16	3836871	2.6
GEVFNAT A1F	2796.0641	25.33	1512501	3.6
GEVFNAT A1(F)2-Gal+GlcNAc	2983.1444	25.34	1320994	4.8
GEVFNAT A1F-Gal	2634.0105	25.51	608358	4.1
GEVFNAT A2F	3087.1629	25.77	805142	2.2
GEVFNAT A1F	2796.0644	25.80	541989	3.5
GEVFNAT A1F-Gal	2634.0125	25.86	3692097	3.4

We observed one hundred and forty glycopeptides by LC–MS. These are recorded grouped by ascending retention time in Supplementary Fig. S1, along with accurate masses, peptide sequences, glycan assignments, and a key to the glycan structures. The location of each glycopeptide series on Spike protein is indicated in the first column. It may be seen that all observed glycans for the same peptide occur within a four-minute retention time window. Accurate mass and estimated retention time are included for a further three hundred and six glycopeptides.

In the pseudo-MS3 experiment, glycans were lost by in-source decay. GEVFNAT-GlcNAc was isolated in the quadrupole and fragmented in the collision cell. Sequence confirmation for the peptide stump GEVFNAT-GlcNAc with mass errors calculated is shown in Supplementary Fig. S2.

**Fig. 4:**
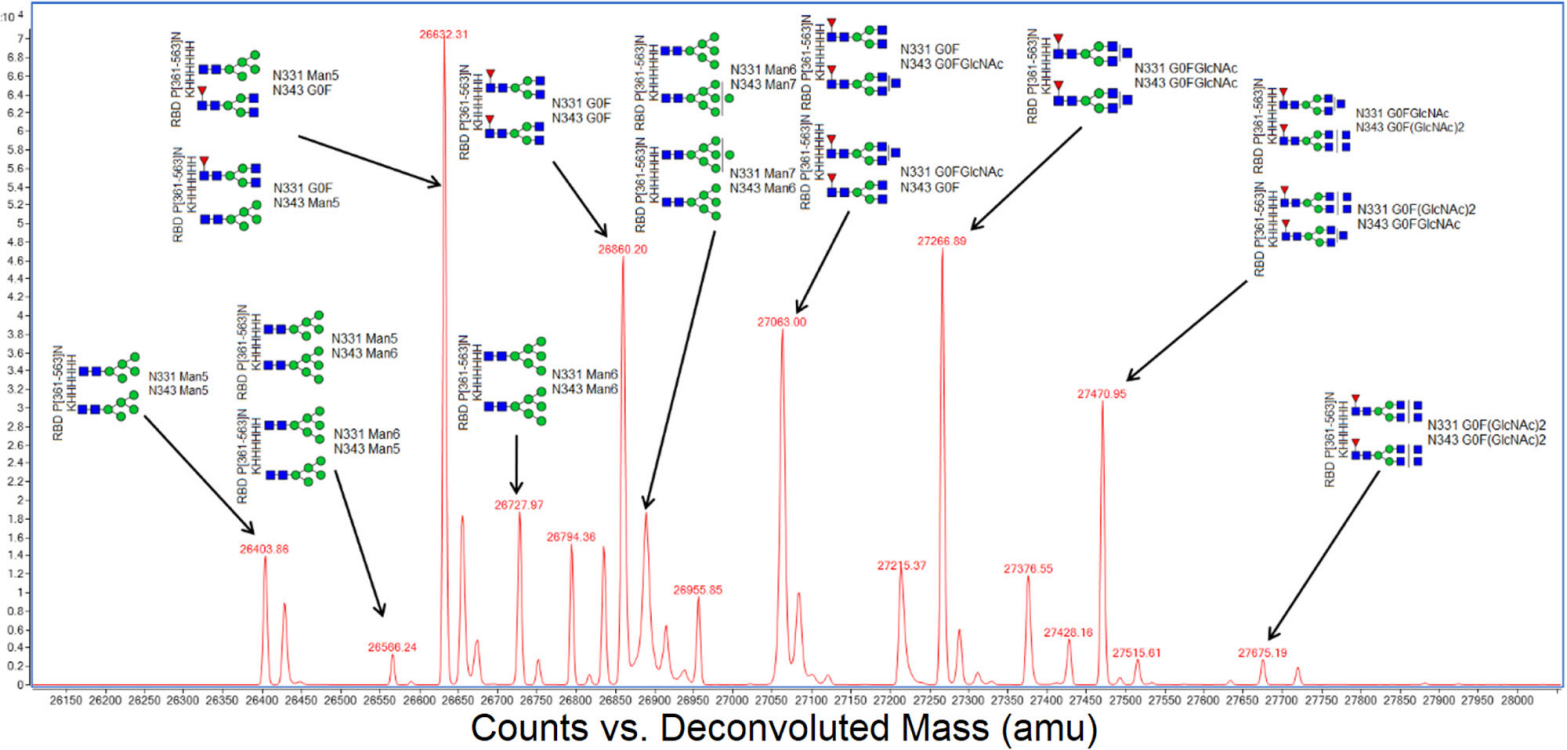
Intact mass analysis of RBD protein. Labels indicate the principal glycan species Man5, G0F, and G0F+GlcNAc occurring at sequence positions N331 and N343, which are in agreement with glycopeptide analysis. Note that this method cannot differentiate individual glycosylation sites; hence, when two structures are possible, both are shown.

Intact mass measurement of fully glycosylated Spike protein was unsuccessful due to the polydispersity of its innumerable glycoforms and the resulting dilution of ion signal. However, the smaller RBD protein, bearing only two glycosylation sites, did prove amenable to intact mass analysis. Figure 4 shows twenty-one glycoforms for intact RBD protein, of which ten major glycoforms could be assigned. This showed that the principal glycan species were Man5, G0F, and G0F+GlcNAc which agreed with the glycopeptide analysis.

Elastase was chosen as a single digestion enzyme because it was judged to give the best chance of generating glycopeptides with a single NxS/T motif, essential for unambiguous glycan mapping. For non-glycosylated Spike protein peptides, elastase generated 63 high-quality MS–MS hits and 26% coverage allowing for five missed cleavages. The same data searched for nonspecific cleavage gave 135 high-quality MS–MS hits and 48% coverage allowing for twenty missed cleavages. Elastase itself contains 2 NxS/T motifs. We therefore prepared elastase only, at 10x, the usual concentration, searched the resulting LC–MS data using the MRTF database as a control, however no hits were found. The Spike protein LC–MS data did contain a small number of elastase autodigestion peptides.

## Discussion

Glycoprotein analysis is difficult. It is either performed in biopharmaceutical laboratories with proprietary expertize of glycan analysis on simple glycoproteins, such as immunoglobulins, or performed by a handful of academic labs with experience of glycan discovery from complex glycoproteins. Many protein researchers choose to ignore it, manipulating cell lines such that they cannot process beyond Man5, or to remove glycans entirely by mutation at the glycosylation motif or enzymatically^10^. While this approach has its merits, it has exposed a serious weakness in analytical capability when faced with a pathogen such as SARS-CoV-2, which potentially evades the immune system using heavy and complex glycosylation.

Glycosylation of Spike protein in Covid-19 patients is likely to be variable and may change over the course of infection^11^. This is difficult to measure due to clinical sample availability, biohazard, and the concentration/extraction required. Thus, no large study has been reported. So what is a desirable glycosylation profile in recombinant Spike protein? The answer depends on the intended use and can be glycoengineered^12^. For example, when used to screen molecular drug candidates, a glycosylation profile similar to Covid-19 patients is probably needed. Hence, the use of mammalian cell lines such as HEK (human) or CHO (rodent). How different cell lines and expression yields affect glycosylation is currently under investigation within our laboratory. When Spike protein is used in in vitro antibody tests, excessive glycosylation could block antibody binding (false negative), whereas insufficient glycosylation may result in misfolding of Spike protein during expression^13^ and again failure of antibody binding (false negative). Indeed, it may be possible to reduce false positives from cross-reacting previous, non-Covid-19 coronavirus infections by engineering the glycosylation. For a vaccine based on Spike protein, excessive glycosylation may “shield” it from the immune system, making it ineffective as an antigen, whereas insufficient glycosylation may cause misfolding and failure to generate antibodies capable of fighting a real infection. Different glycosylation may indeed provoke a stronger immune response. The Expi293F™ GnTI cell line does not have N-acetylglucosaminyltransferase I activity and is unable to produce complex N-glycans. Expression in insect cell lines has also been reported, producing alternative glycosylation^14^. Interestingly, we have observed a number of G0F+GlcNAc glycopeptides that appear to have a bisecting GlcNAc^15^; these have been implicated in immune-response tolerance^16^. Whatever the glycosylation, a biopharmaceutical vaccine derived from Spike protein requires rigorous characterization and quality control including the glycosylation. In addition, recombinant Spike protein can be glycoengineered using a number of glycosidases to remove (PNGase F), trim, or modify the glycans.

We have chosen an approach relying on elastase digestion to generate glycopeptides bearing a single glycan, but with a sufficient number of amino acid residues to enable chromatographic separation by reverse-phase HPLC, as well as confident identification by accurate mass or *de novo* sequencing. Our choice of reverse-phase HPLC has excellent discrimination for short-elastase peptides, whereas glycans show little or no interaction with the column. Thus, species originating from a single glycosylation site with the same peptide sequence but several different glycans, eluted with the same retention time and could be discriminated by mass spectrometry. We used reverse-phase HPLC and MS–MS to characterize as many glycopeptides as possible. Although this required complex and time-consuming data analysis, it needed only be performed once, with the goal of building an accurate mass-retention time MRTF database for all observed Spike protein glycopeptides. Provided the same HPLC column and mobile-phase conditions are used, retention times should not vary significantly. Thus, working in the analytical mode we describe, glycan structure and peptide sequence is assigned confidently, by accurate mass and retention time alone. LC–MS data need only to be searched against the mass-retention time MRTF database, and peak areas recorded, to generate a complete characterization of Spike protein glycans.

Mass and retention-time databases are already commonly used for small molecule screening identification applications from metabolomics, proteomics, and pesticides to drugs of abuse^17–20^. The MRTF database method described here has advantages over other approaches to Spike protein glycan analysis. Previous studies relied upon very expensive equipment and software unavailable in most analytical laboratories. Working in “analytical” mode, all that is necessary is to reproduce the chromatography, hence, our method is a generic one, which can in principle be run using any HPLC coupled to any accurate mass LC–MS instrument and is not restricted to specific proprietary data analysis software. We used PCDL and *Masshunter*, but MRTF database analysis can be performed on any vendor software or manually. Moreover, it demands no specialized expertize in glycobiology, and is thus accessible to many more researchers. Some published methods require multiple specific endoproteases, some of which cannot be readily sourced. Our method uses a single enzyme, elastase, which is inexpensive and widely available. Nor does it rely on glycosidases, which may not work efficiently and do not cleave O-linked glycans.

Our data contain an excess of glycopeptides with the motif (y)nNxS/T. This appears to be a very convenient function of elastase on glycoproteins, because the presence of the motif at the C terminus facilitates *de novo* sequencing. We would be interested to know if this cleavage bias toward the C terminus of the glycan motif is reproducible in other labs and whether it indicates steric hindrance within the elastase-enzyme structure. If such bias is real, then these glycopeptides are less likely to be a false-positive result.

Receptor-binding domain (RBD) protein from Spike protein is of interest in many labs for development of serological tests or neutralizing antibodies. Because the yield of RBD protein was five times higher than Spike protein and more was initially available, we used it for method optimization, and since it bears only two glycosylation sites that are also present on Spike protein, it functioned as a useful model. Consequently, N343 on glycopeptide GEVFNAT is over-represented in our demonstration MRTF database. We consistently observed the same three major glycans (Man5, G0F, and G0F+GluNAc) on this glycopeptide and these were also in agreement with intact mass analysis of RBD protein as shown in Fig. 4. On closer inspection, glycans up to A2F could also be observed at lower levels. We suspect that sufficiently detailed analysis may reveal all possible glycans with low abundance at all available sites. The most important would therefore be the top three to five glycans grouped together to show, for example, high-mannose, hybrid or complex glycans. If the complete complement of Spike protein glycopeptides proves too challenging for a single analysis, this site N343, which is the most complete, would make a good proxy for total Spike protein glycosylation.

We acknowledge that the mass-retention time fingerprinting MRTF database method described, like all database searching methods, is dependent on the reproducibility of the enzyme digestion and both the quality and the completeness of the database being searched. The example MRTF database reported here is provided as a demonstration. Due to glycan complexity and the likely absence of specific glycans within the Spike protein batches prepared by us, it will always be incomplete. Moreover, individual glycopeptides were identified with variable degrees of certainty, and we recommend that they should be validated by the user. As with all glycan-analysis methods, there is a bias toward glycopeptides that are easiest to identify by the techniques used, and such bias will also be reflected within the MRTF database. Once the MRTF database has been created, it must be refined and extended over time to improve data quality, and it is our intention to do so.

## Methods

### Cloning, expression, and purification of Spike protein

The gene encoding amino acids 1–1208 of the SARS-CoV-2 Spike glycoprotein ectodomain (S), with mutations of RRAR > GSAS at residues 682–685 (to remove the furin cleavage site) and KV > PP at residues 986–987 (to stabilize the protein), was synthesized with a C-terminal T4 fibritin trimerization domain, HRV 3 C cleavage site, 8xHis tag, and Twin-Strep tag^5^. The construct was subcloned into pHL-sec^21^ using the AgeI and XhoI restriction sites and the sequence was confirmed by sequencing. Recombinant Spike protein was produced in *Expi293F*^™^ cells by transient transfection with purified DNA (0.5 mg/L cells) using a 1:6 DNA:L-PEI ratio, mixed in minimal medium, and sodium butyrate as an additive. Cells were grown in suspension in *FreeStyle293*^™^ medium with shaking at 150 rpm in 2 L of smooth roller bottles, filled with 0.5 L cells at 2 e^6^/mL per bottle at 30 °C with 8% CO_2_ and 75% humidity. Supernatants from transfected cells were harvested three days post transfection by centrifugation. Clarified supernatant was mixed with Ni^2+^ IMAC *Sepharose*^®^ *6 Fast Flow* (*GE*; 2 mL of bed volume per L of supernatant) at room temperature for 2 h. Using a gravity-flow column, resin was collected and washed stringently with 50 CV each of base buffer (1X PBS), WB25 (BB+ 25 mM imidazole), and WB40 (BB+ 40 mM imidazole), followed by elution with EB (0.30 M imidazole in 1X PBS). Protein was dialyzed into 1X PBS using *SnakeSkin*™ 3500 MWCO dialysis tubing, concentrated to 1 mg/mL using a 100,000 MWCO *VivaSpin* centrifugal concentrator (*GE*), and centrifuged at 21,000 × *g* for 30 min to remove aggregates. The trimeric Spike protein was flash-frozen in LN_2_ and stored at −80 °C until use. The final purified yield was 1 mg of Spike protein per L of transfected cells.

### Cloning, expression, and purification of receptor binding domain

The receptor-binding domain (RBD; aa 330–532) of SARS-CoV-2 Spike protein (Genbank MN908947) was inserted into the pOPINTTGneo expression vector fused to an N-terminal signal peptide and a C-terminal 6xHis tag^22^. RBD protein was produced by transient transfection in *Expi293F*™ cells (*ThermoFisher Scientific*, UK) using purified DNA (1.0 mg/L cells), a 1:3 DNA:L-PEI ratio, and sodium butyrate as an additive. Cells were grown in suspension in *FreeStyle293*™ expression medium at 37 °C with 8% CO_2_ and 75% humidity. Supernatants from transfected cells were harvested three days post transfection and the supernatant was collected by centrifugation. Clarified supernatant was incubated with 5 mL of Ni^2+^ IMAC *Sepharose*^®^ *6 Fast Flow* (*GE*) at room temperature for 2 h. Using gravity flow, resin was washed with 50 CV of base buffer (1X PBS) and 50 CV of WB (1X PBS + 25 mM imidazole) before elution with EB (0.5 M imidazole in 1X PBS). Protein was concentrated using a 10,000-MWCO *Amicon Ultra-15* before application to *a Superdex 75* 16/600 column pre-equilibrated with 1X PBS pH 7.4. Peak monomeric fractions were pooled and concentrated to 2 mg/mL, flash-frozen in LN_2_, and stored at −80 °C until use. The final purified yield was >15 mg of RBD protein per L of transfected cells.

### Sample preparation

SARS-CoV-2 Spike protein or RBD-6H protein at 1 mg/mL in PBS were prepared in aliquots of either 20 µL or 80 µL and diluted 1 in 3 in 100 mM ammonium bicarbonate, pH 8.0, followed by reduction by addition of 1, 4 dithiothreitol (DTT) to 5 mM and incubation at 37 °C for 1 h. Next, the protein was alkylated by addition of iodoacetamide (IAA) to 15 mM and incubation in the dark for 30 min. This was followed by overnight digestion using elastase (*Promega*) at a ratio of 1:20 (w/w). The following day, the supernatant was dried using a rotary evaporator, and resuspended in 60 µL of 0.1% formic acid for injection into the LC–MS. The use of 6 M urea was found to be unnecessary and reduced signal.

### ‘Analytical mode’ LC–MS glycopeptide data acquisition

LC–MS “analytical mode” was performed using a *1290 Infinity* UHPLC coupled to a *G6530A* ESI QTOF mass spectrometer (*Agilent Technologies*). TOF and quadrupole were calibrated prior to analysis and the reference ion 922.0098 *m/z* was used for continuous mass correction. The sample was introduced using a 50 µL full-loop injection. Reverse-phase (RP) chromatographic separation was achieved using *an AdvancedBio Peptide* reverse-phase 2.7 µm particle, 2.1 mm×100 mm column (655750-902 *Agilent Technologies*). Mobile phase A was 0.1% formic acid in water and mobile phase B 0.1% formic acid in methanol (*Optima* LC–MS grade, *Fisher*), acetonitrile was found to reduce the signal. Initial conditions were 5% B and 0.200 mL/min flow rate. A linear gradient from 5% B to 60% B was applied over 60 min, followed by isocratic elution at 100% B for 2 min returning to initial conditions for a further 2 min. Post time was 10 min to re-equilibrate the column. A blank was run between each sample to monitor possible carryover/precipitation on the column. MS-source parameters were drying-gas temperature 350 °C, drying-gas flow 8 L/min, nebulizer pressure 30 psi, capillary 4000 V, and fragmentor 150 V. MS spectrum range was 100–3200 *m/z* (centroid only), 2-GHz extended dynamic range, with the instrument in positive-ion mode.

Any high-resolving reverse-phase column could be used, but the retention times may need to be adjusted. We considered the following in column selection: C18 superficially porous particle of 2.7 μm, pore size 120 Å, and low-flow rate gave good chromatographic resolution while keeping the back pressure below 300 bar (no need for a UHPLC); a small internal column diameter of 2.1 mm reduces dilution and a length of 100 mm gave good chromatographic resolution within a reasonable run time. A simple gradient was applied free of inflection points for robustness of MRTF database retention-time matching.

Reverse-phase chromatography separates peptides based on their accessible hydrophobic groups to the partitioning mobile and stationary phases of the column, peptide amphipathic secondary α-helical structure complicates the interaction^10^. Elastase digestion of Spike protein produced specific, “nonspecific”, and missed cleavage peptides, reproducibly, along the primary amino acid sequence^14^. Hence, elastase was treated as a non-specific protease for discovery workflows. This makes the use of in silico prediction of retention times^23^ difficult because we could not be sure a priori which peptides were going to be produced without running the sample.

### LC–MS–MS glycopeptide data-acquisition “discovery mode”

LC–MS–MS “discovery mode” was performed as described above, with the following changes: soft CID collision energy parameters for MS–MS were slope 1.0, intercept 0 using argon as the collision gas (if using nitrogen slope 1.5, intercept 0) was used to favor glycan fragmentation over peptide fragmentation for glycopeptides. Sufficient non-glycosylated peptides were fragmented to give reasonable sequence coverage. Care was taken to reduce sodium and potassium contamination where possible and Tris buffers were avoided as these adducts interfere with glycopeptide analysis.

### LC–MS glycopeptide data analysis “analytical mode”

Analysis only requires retention time and accurate mass data using the Spike protein MRTF database created as described below. This is possible using the *Agilent* software described, software provided by other vendors, or by manual inspection. In our case, we used *Masshunter Qualitative Analysis* version B.07 (*Agilent Technologies*) and the Molecular Feature Extraction tool to extract H^+^, Na^+^, and K^+^ adducts and charge states +1 to +5. Briefly, this tool identifies and associates common spectral features such as carbon isotopes, adducts and multiple charge states as belonging to the same compound (peptide) by virtue of sharing the same accurate mass and retention time, then combines these features together to give a mass, retention time, and volume for each compound. Compounds were then searched against Spike protein MRTF database using a mass error window +/−10 ppm and a retention time window +/−2 min. Some filtering of the data was used to reduce the number of compounds and hence speed up the database search. Relative quantitation of each glycan on a particular glycopeptide could then be assessed.

### LC–MS–MS glycopeptide analysis “discovery mode”

Construction of the Spike protein glycopeptide MRTF database (“discovery mode”) was more complex and time-consuming, but once constructed and made available to the scientific community, there is no further need to repeat this step. By using reverse-phase HPLC, glycopeptides are separated by the relatively hydrophobic peptide moiety, whereas the associated hydrophilic glycans are grouped together by retention time as illustrated in Supplementary Fig. S3.

Initial LC–MS–MS discovery mode data for incorporation into a glycopeptide MRTF were performed using *Masshunter Qualitative Analysis* with *Bioconfirm* B.07.00 (*Agilent Technologies*). Compounds were identified using the Find by Molecular Feature (MFE) tool looking for H^+^, Na^+^, and K^+^ adducts and charge states +2 to +5. The results were filtered to remove compounds <1000 Da (too small to be glycopeptides). Compound MS–MS spectra were screened manually for the following oxonium reporter ions: Hex *m/z* 163.0601, HexNAc *m/z* 204.0866, HexHexNAc *m/z* 366.1395, Neu5Ac *m/z* 274.0921/*m/z* 291.0949, and/or a Hexose ladder _δ_M 162.0528 Da. High-quality *m/z* spectra were deconvoluted to neutral mass spectra with glycan *de novo* interpretation performed manually. Once a glycopeptide had been identified, it was entered into a personal compound data library database (PCDL, *Agilent Technologies*) as a mass and retention time. In addition, the database made use of known mammalian N-linked glycan processing. After the initial glycopeptide identification, other processed glycopeptides, which were considered likely to also be present, were added to the database at the same retention time and with a calculated mass. For example, if a glycopeptide with Man5 was identified by MS–MS, Man1–9 and G0/F were added at the same retention time. If these glycans were subsequently found in the data, their actual retention times were updated, and the next round of processing to more complex glycans was added, in order to produce the most comprehensive MRTF database possible, while still being manageable. Processing order:

$${{{{{\rm{Man}}}}}}({{{{{\rm{n}}}}}})\to {{{{{\rm{G}}}}}}0/{{{{{\rm{F}}}}}}\to {{{{{\rm{G}}}}}}1/{{{{{\rm{F}}}}}}\to {{{{{\rm{G}}}}}}2/{{{{{\rm{F}}}}}}\to {{{{{\rm{A}}}}}}1/{{{{{\rm{F}}}}}}\to {{{{{\rm{A}}}}}}2/{{{{{\rm{F}}}}}}\to {{{{{\rm{Very}}}}}}\,{{{{{\rm{Complex}}}}}}$$

**Fig. 5:**
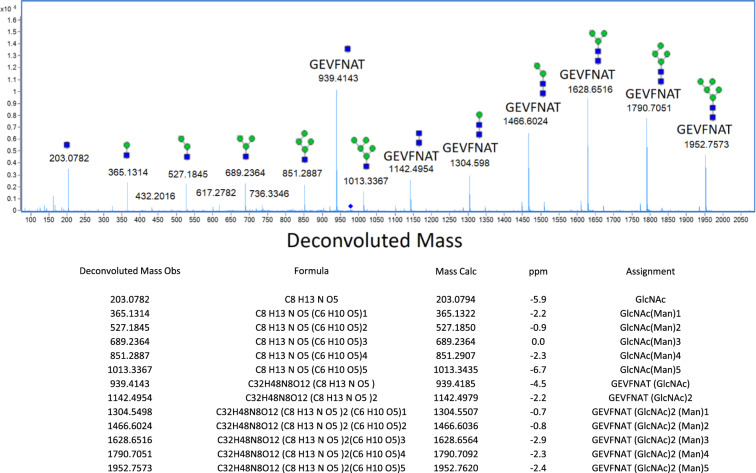
Complete glycan fragmentation series for RBD glycopeptide GEVFNAT-Man5 (N343). Complete glycan fragmentation series for RBD glycopeptide GEVFNAT-Man5 (N343) with calculated mass errors. Glycan-fragmentation series shows the peptide stump (GEVFNAT-GlcNAc) and mannose ladders. Blue squares indicate N-acetyl glucosamine, green circles indicate mannose.

Valid glycan identifications resulted in a calculated peptide mass that could be matched to the sequence. Where high-quality spectra were present, a peptide-GlcNAc stump was observed (Fig. 5). This was used in a pseudo-MS3 experiment with manual peptide *de novo* interpretation to confirm the peptide sequence (supplementary data Fig. S1). Mass data adjacent to the glycopeptide-retention time were then searched for neutral differences corresponding to glycans, for example, Man5 → G0F or Man7 → G2F has a neutral delta mass of 228.1111 Da.

Data for the most likely glycan were added to the MRTF database, including a deconvoluted mass MSMS spectra were available, using nomenclature generating the most easily readable format. As expected, not all species could be matched to the sequence, presumably due to unexpected modifications or possibly O-linked glycopeptides. In this case, they were added to the MRTF database as “GP” with an identifying number and with as much information as possible which could be extracted.

A second round of glycopeptide discovery used *Bioconfirm* v10.0 data analysis software (*Agilent Technologies*). Sequences were matched by peptide accurate mass using the following parameters: peptide cleavage nonspecific, number of missed cleavages 20, and N-linked modifications Man3, Man5–9, G0, G0F, G0F GlcNAc, G1, G1F, G2, and G2F. Any peptide bearing the glycosylation motif NxS/T with two or more glycan hits within a retention-time window +/−2 min was added to the MRTF database, except missed cysteine alkylations.

In-source fragmentation due to glycopeptide ions absorbing excess energy could be identified in the MS by searching extracted-ion chromatograms (EICs) of the oxonium reporter ions and also by related glycopeptides appearing with exactly the same retention times. Both were observed infrequently and at manageable levels.

### Intact mass analysis of RBD protein

Concentrated protein samples were diluted to 0.02 mg/mL in 0.1% formic acid and 50 µL was injected on to a 2.1 × 12.5 mm *Zorbax* 5 µm *300SB-C3* guard column (*Agilent Technologies*) housed in a column oven set at 40 °C. The solvent system used consisted of 0.1% formic acid (solvent A) and 0.1% formic acid in methanol (solvent B). Chromatography was performed as follows: initial conditions were 90% A and 10% B and a flow rate of 1.0 mL/min. A linear gradient from 10% B to 80% B was applied over 35 s. Elution then proceeded isocratically at 95% B for 40 s, followed by equilibration at initial conditions for a further 15 s. The mass spectrometer was configured with the standard ESI source and operated in positive-ion mode. The ion source was operated with the capillary voltage at 4000 V, nebulizer pressure at 60 psig, drying gas at 350 °C, and drying-gas flow rate at 12 L/min. The instrument ion-optic voltages were as follows: fragmentor 250 V, skimmer 60 V, and octopole RF 250 V.

### Statistics and reproducibility

The complexity of the data presented a challenge both in analysis and presentation, all options introduce a bias, especially data at the threshold of detection. The sensitivity of our LC QTOF instrument that dictated at least 80 µg of Spike protein (or 15 µg of RBD) was required to give reasonable data for the most abundant glycopeptides. Injection of more would be likely to result in improved data quality, which might appear excessive for nano-ESI proteomic methodology, but is reasonable in a biopharmaceutical production QC context, where the sample is less limited. We chose three samples with different expression parameters as examples of “diverse” biological replicates, performed a single-elastase digestion on each, and then injected the same sample 3 times as a pure LC QTOF system technical replicate.

$${{{{{\rm{GSK}}}}}}-{{{{{\rm{CHO}}}}}}\_{{{{{\rm{CVSA}}}}}}-{{{{{\rm{p}}}}}}095\_80\,{\rm \mu g}:\,{{{{{\rm{technical}}}}}}\,{{{{{\rm{replicates}}}}}}\,{{{{{\rm{A}}}}}},\,{{{{{\rm{B}}}}}}\,\& \,{{{{{\rm{C}}}}}}$$

$${{{{{\rm{SPIKE}}}}}}16\_{{{{{\rm{transient}}}}}}\_{{{{{\rm{CVSA}}}}}}-{{{{{\rm{p}}}}}}046\_80\,{\rm \mu {{{{{\rm{g}}}}}}}:\,{{{{{\rm{technical}}}}}}\,{{{{{\rm{replicates}}}}}}\,{{{{{\rm{A}}}}}},\,{{{{{\rm{B}}}}}}\,\& \,{{{{{\rm{C}}}}}}$$

$${{{{{\rm{SPIKE}}}}}}23\_{{{{{\rm{stable}}}}}}\_{{{{{\rm{CVSA}}}}}}\_80\,{\rm \mu {{{{{\rm{g}}}}}}}:\,{{{{{\rm{technical}}}}}}\,{{{{{\rm{replicates}}}}}}\,{{{{{\rm{A}}}}}},\,{{{{{\rm{B}}}}}}\,\& \,{{{{{\rm{C}}}}}}$$

This gave good data for seven distinct sites and partial data for a number of others. As expected, the technical replicates were very close, as shown in Supplementary Fig. S4. We chose abundance bar charts to make comparison with different batches more informative, because important information is lost in pie charts, which give the impression of equal expression. It is our experience that zero, null values, and noise can play havoc in statistically driven software, and hence care was taken to check the raw data. In the case of GSK-CHO B, glycopeptide GEVFNAT A2F is missing where the molecular feature-extraction algorithm failed to extract the data.

In the absence of a gravimetrically weighted internal standard for absolute quantitation using a standard curve, we relied on relative quantitation based on the relative abundance of the glycopeptides, and made the following assumptions: (i) the relative abundance of each glycan on the same peptide was proportional to its concentration (probably true), (ii) the relative abundance of different peptides was proportional to their concentration and /or importance (may not be true), and (iii) all biologically important glycopeptides give a response (unknown). We saw no evidence in the form of good auto-MS-MS data for glycan-free peptides for any of the 22 possible NxS/T motifs. Concluding fully occupied sites from this, however, would be over-interpretation and more work is required.

Comparison between our “diverse” biological replicates was more complex. The overall pattern of glycosylation looked similar and ratios within sites, high mannose, Man5/G0F, and complex sites, were similar across all 3 biological replicates. The GSK-CHO and SPIKE23 were the most similar with the absolute abundances of the GSK-CHO glycans on average x 1.5 that of the other. Interestingly, no G0F+GlcNAc glycans were found in the GSK-CHO data. GSK-CHO and SPIKE23 compared to the SPIKE16 show much greater variation in absolute abundance with some sites the same, some half, and some double the abundance. The GEVFNAT site in the SPIKE16 gave no results, but we believe this is because it dipped below the limit of detection rather than the site is non-glycosylated, especially as glycopeptide NLCPFGEVFNAT was found in some abundance.

To try and resolve whether the observed differences were due to differences in expression, incorrect protein amount, protein concentration (reaction kinetics), or enzyme digestion we looked at 17 non-glycosylated peptides that gave good identifications by MS–MS and were present in all samples. (The possible effect of Spike glycosylation upon elastase digestion itself should also be considered, and we believe that it may have an effect.) These non-glycosylated peptides showed even greater variation than the glycopeptides, sometimes by an order of magnitude. As the source of the variation was uncertain, we chose to present the data as simple bar charts of abundance, rather than normalize the data, and leave interpretation to the analyst. An example is given in supplementary Fig. S4.

### Reporting summary

Further information on research design is available in the Nature Research Reporting Summary linked to this article.

## Supplementary information

Transparent Peer Review File

Supplementary Information

Reporting Summary

## Data Availability

The complete Spike protein PCDL (Personal Compound Database Library, *Agilent Technologies*) database is available to download in.cdb or.xlsx format here: https://zenodo.org/record/3958218#.Xxn_BChKhoY. 10.5281/zenodo.3958218^24^. Source data are available here or from the corresponding author upon reasonable request. https://zenodo.org/record/4911578#.YNxyxufTVaQ. 10.5281/zenodo.4911578^25^. Any remaining information can be obtained from the corresponding author upon reasonable request.
