# Supplementary material for: Identification, mapping and relative quantitation of SARS-CoV-2 Spike glycopeptides by Mass-Retention Time Fingerprinting
Source: Commun Biol. 2021 Aug 3;4:934. doi: 10.1038/s42003-021-02455-w (PMC8333269; doi:10.1038/s42003-021-02455-w)
Supplement: Supplementary file 2 — Supplementary Information [file 42003_2021_2455_MOESM2_ESM.pptx]

## Slide 1
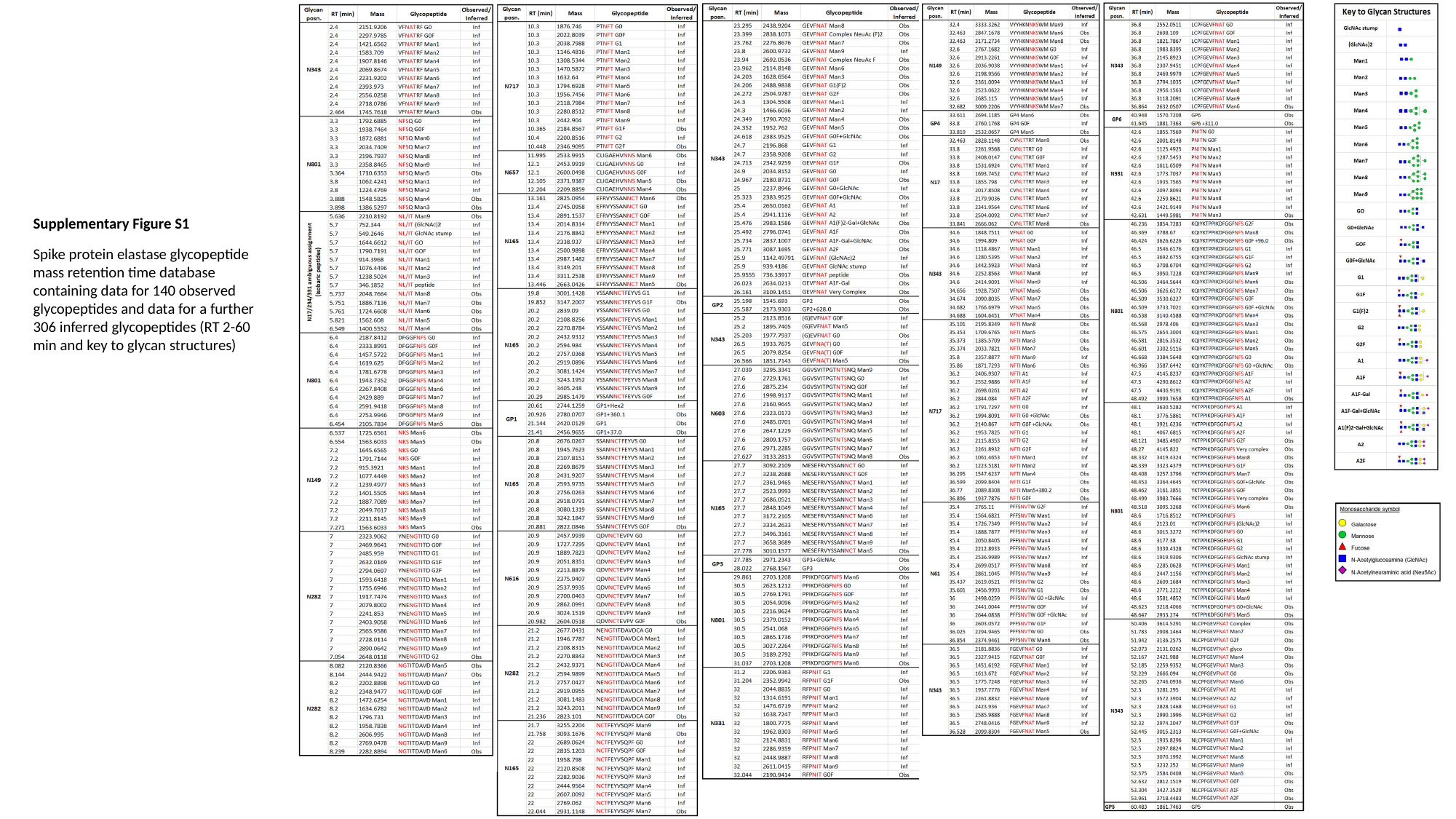

Supplementary Figure S1
Spike protein elastase glycopeptide mass retention time database containing data for 140 observed  glycopeptides and data for a further 306 inferred glycopeptides (RT 2-60 min and key to glycan structures)

## Slide 2
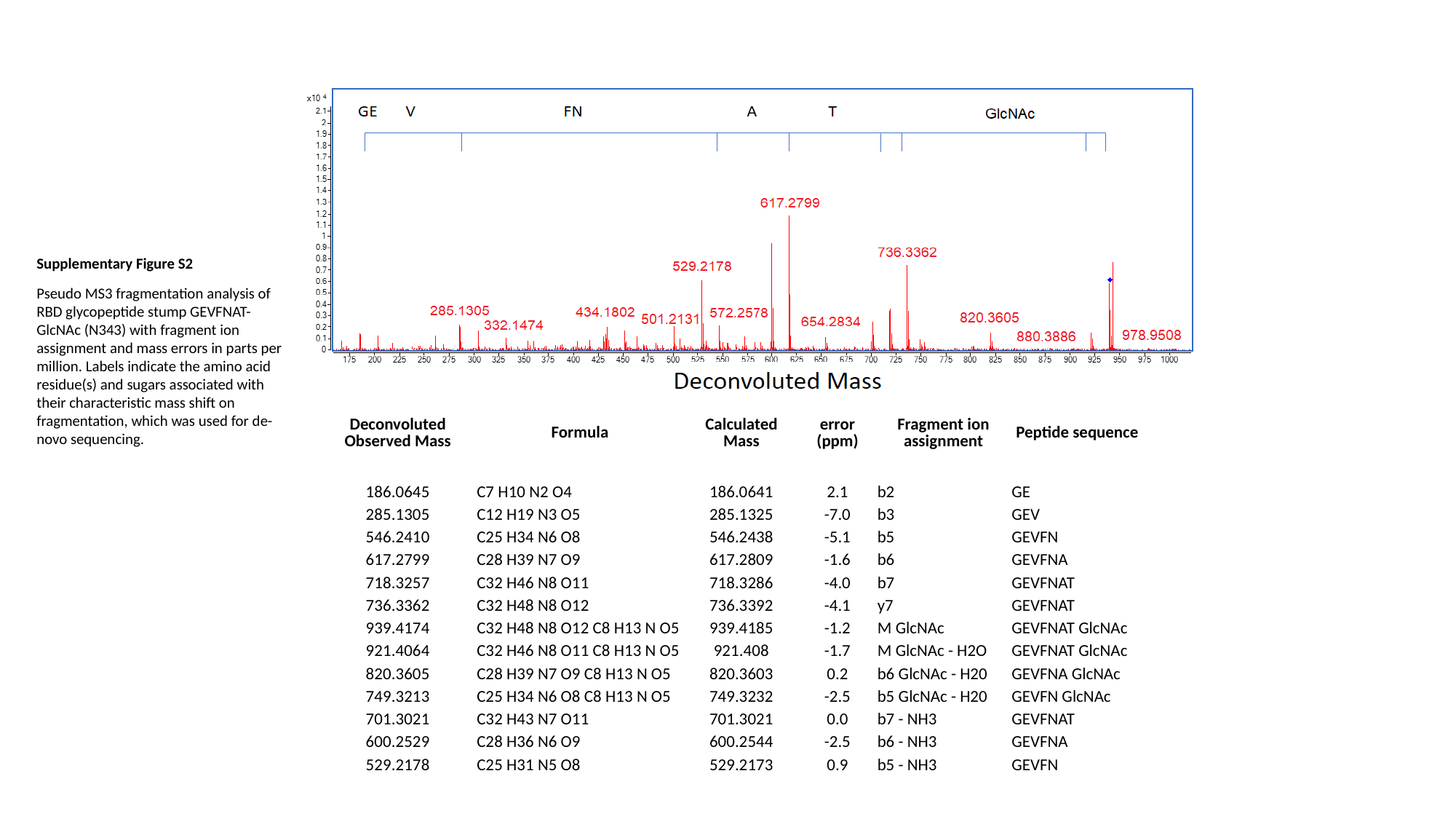

Supplementary Figure S2
Pseudo MS3 fragmentation analysis of RBD glycopeptide stump GEVFNAT-GlcNAc (N343) with fragment ion assignment and mass errors in parts per million. Labels indicate the amino acid residue(s) and sugars associated with their characteristic mass shift on fragmentation, which was used for de-novo sequencing.
| Deconvoluted Observed Mass | Formula | Calculated Mass | error (ppm) | Fragment ion assignment | Peptide sequence |
| --- | --- | --- | --- | --- | --- |
| | | | | | |
| 186.0645 | C7 H10 N2 O4 | 186.0641 | 2.1 | b2 | GE |
| 285.1305 | C12 H19 N3 O5 | 285.1325 | -7.0 | b3 | GEV |
| 546.2410 | C25 H34 N6 O8 | 546.2438 | -5.1 | b5 | GEVFN |
| 617.2799 | C28 H39 N7 O9 | 617.2809 | -1.6 | b6 | GEVFNA |
| 718.3257 | C32 H46 N8 O11 | 718.3286 | -4.0 | b7 | GEVFNAT |
| 736.3362 | C32 H48 N8 O12 | 736.3392 | -4.1 | y7 | GEVFNAT |
| 939.4174 | C32 H48 N8 O12 C8 H13 N O5 | 939.4185 | -1.2 | M GlcNAc | GEVFNAT GlcNAc |
| 921.4064 | C32 H46 N8 O11 C8 H13 N O5 | 921.408 | -1.7 | M GlcNAc - H2O | GEVFNAT GlcNAc |
| 820.3605 | C28 H39 N7 O9 C8 H13 N O5 | 820.3603 | 0.2 | b6 GlcNAc - H20 | GEVFNA GlcNAc |
| 749.3213 | C25 H34 N6 O8 C8 H13 N O5 | 749.3232 | -2.5 | b5 GlcNAc - H20 | GEVFN GlcNAc |
| 701.3021 | C32 H43 N7 O11 | 701.3021 | 0.0 | b7 - NH3 | GEVFNAT |
| 600.2529 | C28 H36 N6 O9 | 600.2544 | -2.5 | b6 - NH3 | GEVFNA |
| 529.2178 | C25 H31 N5 O8 | 529.2173 | 0.9 | b5 - NH3 | GEVFN |

## Slide 3
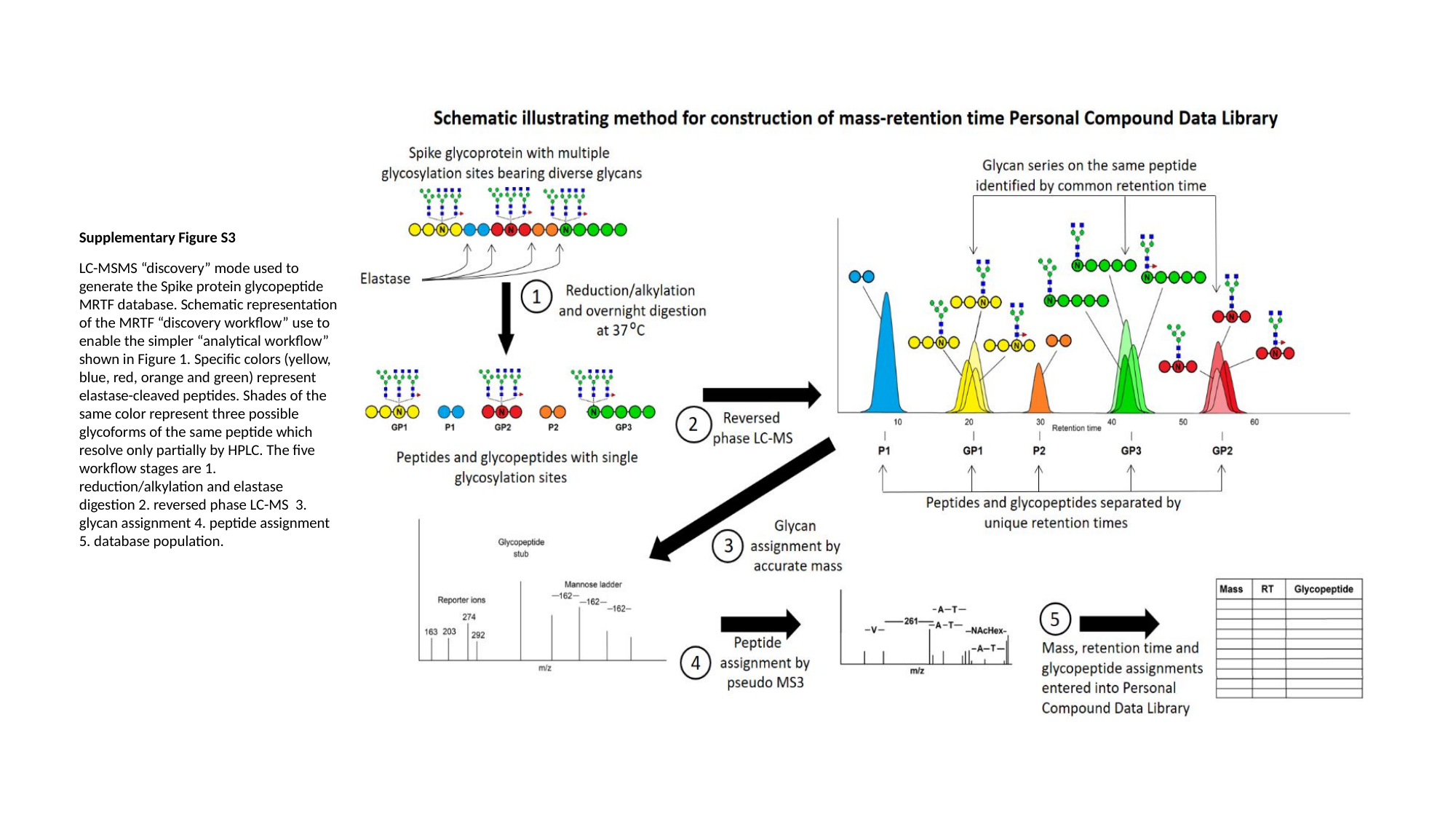

Supplementary Figure S3
LC-MSMS “discovery” mode used to generate the Spike protein glycopeptide MRTF database. Schematic representation of the MRTF “discovery workflow” use to enable the simpler “analytical workflow” shown in Figure 1. Specific colors (yellow, blue, red, orange and green) represent elastase-cleaved peptides. Shades of the same color represent three possible glycoforms of the same peptide which resolve only partially by HPLC. The five workflow stages are 1. reduction/alkylation and elastase digestion 2. reversed phase LC-MS 3. glycan assignment 4. peptide assignment 5. database population.

## Slide 4
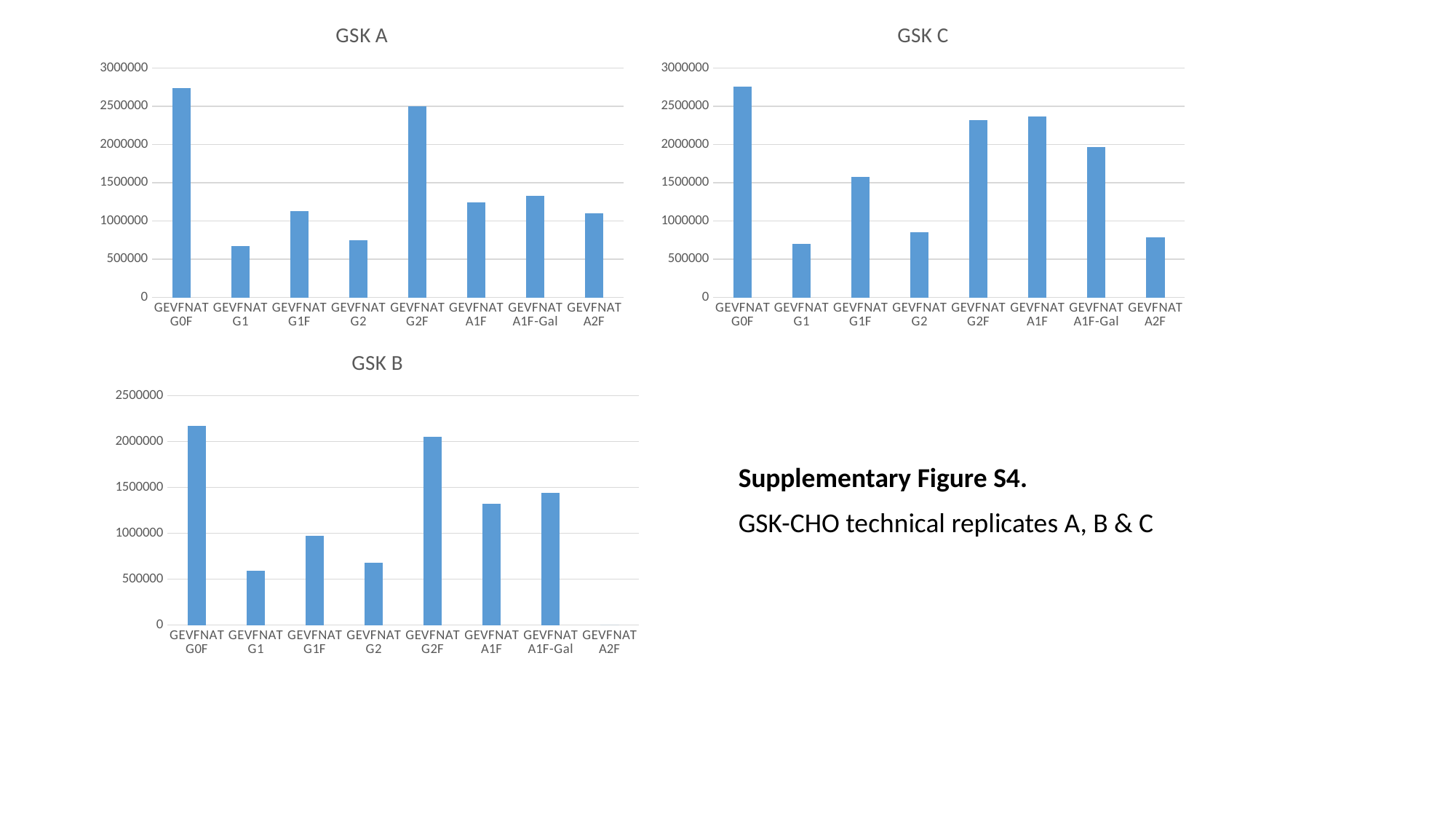

### Chart: GSK A
| Category | |
|---|---|
| GEVFNAT G0F | 2741598.0 |
| GEVFNAT G1 | 677284.0 |
| GEVFNAT G1F | 1129632.0 |
| GEVFNAT G2 | 747877.0 |
| GEVFNAT G2F | 2502975.0 |
| GEVFNAT A1F | 1242706.0 |
| GEVFNAT A1F-Gal | 1325241.0 |
| GEVFNAT A2F | 1096655.0 |
### Chart: GSK C
| Category | |
|---|---|
| GEVFNAT G0F | 2755161.0 |
| GEVFNAT G1 | 700121.0 |
| GEVFNAT G1F | 1579297.0 |
| GEVFNAT G2 | 856165.0 |
| GEVFNAT G2F | 2324828.0 |
| GEVFNAT A1F | 2364160.0 |
| GEVFNAT A1F-Gal | 1965104.0 |
| GEVFNAT A2F | 788888.0 |
### Chart: GSK B
| Category | |
|---|---|
| GEVFNAT G0F | 2168903.0 |
| GEVFNAT G1 | 588799.0 |
| GEVFNAT G1F | 973086.0 |
| GEVFNAT G2 | 679945.0 |
| GEVFNAT G2F | 2049471.0 |
| GEVFNAT A1F | 1318771.0 |
| GEVFNAT A1F-Gal | 1442069.0 |
| GEVFNAT A2F | 0.0 |Supplementary Figure S4.
GSK-CHO technical replicates A, B & C

## Slide 5
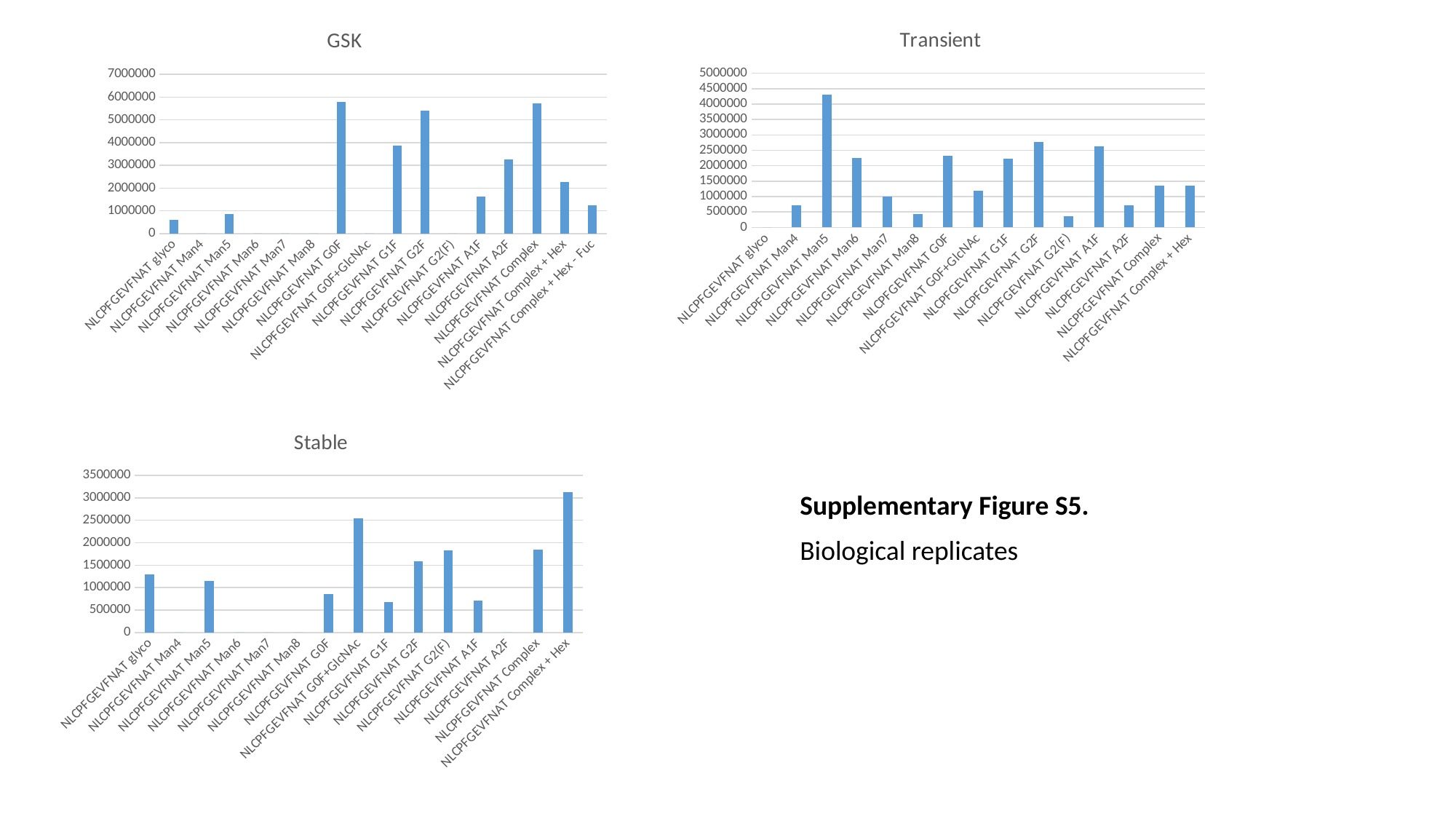

### Chart: GSK
| Category | |
|---|---|
| NLCPFGEVFNAT glyco | 612382.0 |
| NLCPFGEVFNAT Man4 | 0.0 |
| NLCPFGEVFNAT Man5 | 868004.0 |
| NLCPFGEVFNAT Man6 | 0.0 |
| NLCPFGEVFNAT Man7 | 0.0 |
| NLCPFGEVFNAT Man8 | 0.0 |
| NLCPFGEVFNAT G0F | 5797937.0 |
| NLCPFGEVFNAT G0F+GlcNAc | 0.0 |
| NLCPFGEVFNAT G1F | 3869392.0 |
| NLCPFGEVFNAT G2F | 5401840.0 |
| NLCPFGEVFNAT G2(F) | 0.0 |
| NLCPFGEVFNAT A1F | 1631877.0 |
| NLCPFGEVFNAT A2F | 3248884.0 |
| NLCPFGEVFNAT Complex | 5730950.0 |
| NLCPFGEVFNAT Complex + Hex | 2273681.0 |
| NLCPFGEVFNAT Complex + Hex - Fuc | 1256978.0 |
### Chart: Transient
| Category | |
|---|---|
| NLCPFGEVFNAT glyco | 0.0 |
| NLCPFGEVFNAT Man4 | 714701.0 |
| NLCPFGEVFNAT Man5 | 4311466.0 |
| NLCPFGEVFNAT Man6 | 2252216.0 |
| NLCPFGEVFNAT Man7 | 1003091.0 |
| NLCPFGEVFNAT Man8 | 438964.0 |
| NLCPFGEVFNAT G0F | 2329768.0 |
| NLCPFGEVFNAT G0F+GlcNAc | 1193820.0 |
| NLCPFGEVFNAT G1F | 2223761.0 |
| NLCPFGEVFNAT G2F | 2770397.0 |
| NLCPFGEVFNAT G2(F) | 371276.0 |
| NLCPFGEVFNAT A1F | 2619537.0 |
| NLCPFGEVFNAT A2F | 710884.0 |
| NLCPFGEVFNAT Complex | 1350958.0 |
| NLCPFGEVFNAT Complex + Hex | 1363507.0 |
### Chart: Stable
| Category | |
|---|---|
| NLCPFGEVFNAT glyco | 1301336.0 |
| NLCPFGEVFNAT Man4 | 0.0 |
| NLCPFGEVFNAT Man5 | 1142345.0 |
| NLCPFGEVFNAT Man6 | 0.0 |
| NLCPFGEVFNAT Man7 | 0.0 |
| NLCPFGEVFNAT Man8 | 0.0 |
| NLCPFGEVFNAT G0F | 856188.0 |
| NLCPFGEVFNAT G0F+GlcNAc | 2544013.0 |
| NLCPFGEVFNAT G1F | 679594.0 |
| NLCPFGEVFNAT G2F | 1580961.0 |
| NLCPFGEVFNAT G2(F) | 1832127.0 |
| NLCPFGEVFNAT A1F | 709857.0 |
| NLCPFGEVFNAT A2F | 0.0 |
| NLCPFGEVFNAT Complex | 1847585.0 |
| NLCPFGEVFNAT Complex + Hex | 3121783.0 |Supplementary Figure S5.
Biological replicates
