# Supplementary material for: Identification, mapping and relative quantitation of SARS-CoV-2 Spike glycopeptides by Mass-Retention Time Fingerprinting
Source: Commun Biol. 2021 Aug 3;4:934. doi: 10.1038/s42003-021-02455-w (PMC8333269; doi:10.1038/s42003-021-02455-w)
Supplement: Supplementary file 1 — Transparent Peer Review File [file 42003_2021_2455_MOESM1_ESM.pdf]

Reviewers' comments:

Reviewer #2 (Remarks to the Author):

The paper overall is well-organized and very informative. The idea of using the accurate mass and retention time as the fingerprint for data matching has been implemented in the last few decades for -omics analysis(Ex: https://link.springer.com/article/10.1007/s00216-019-01997-7, and https://pubmed.ncbi.nlm.nih.gov/15801753/), and particularly glycopeptides. However, this is one of the pioneer papers on the application of this approach on SARS-COV-2 Spike, which is novel and beneficial for the other biomedical research related to SARS-COV-2. First, the instrumentations used in this paper are easy to adapt to other generic labs. By using the LC-MS approach for their "analytical approach," it makes the process user friendly to a variety of lab, instead of being limited to nano-LC, which is more expensive and high-maintain. Second, the method is straightforward and already utilized in a different type of RPLC column. It is easy to transfer from lab to lab. Both features, together with the database, support the ideal of the paper to promote this database as the universal database for any application using this particular column and gradients. The paper described precisely the method, in particular, the authors made a few interesting remarks of the data in the discussion section about the appearance of the abundant peptides as well as the low abundant glycan. It would be more meaningful if the authors can relate the observations to the biology of SARS-COV-2. On the other hand, because of the generality of the retention time-accurate mass database and RPLC gradient used in the paper, it would be better to see the authors cited a few articles about the realistic ideas of this type of database. Scientifically, it is also better to mention the advantages of using this column compare to other known columns to convince other users for the investment. Moreover, for the data analysis, can the authors observe any trend of elutions based on different chemical differences of the glycopeptides? And if feasible, can the author present this relation using the linear regression to further making the in-silico prediction for the retention time feature of the library/database. Moreover, there are some terms in the paper needed more clarifications or explanations in the results section to help to paper to reach a wide range of readers.

Reviewer #3 (Remarks to the Author):

Excellent concise and descriptive title. This method is straight forward and has high potential as a rapid and invaluable tool in detailed analysis of glycosylation moieties on many proteins of clinical significance.

I have made a few stylist suggestion as I believe an impassive tone is needed in places and care must be taken not to overstate.

Abstract

Remove "rapid and robust identification" there is insufficient evidence to substantiate this claim. Similarly no evidence that "any LC-ToF" can use this technique. Although it has huge potential these claims can only be made once the technique has been replicated by independent laboratories.

Also define typical mass spectrometry laboratory, as clinical microbiology mass spectrometry laboratories do not use LC-MS but use exclusively MALDI-Tofs.

"We stress, however, that there is no " delete We stress as this is not needed starting "However" makes the point and does do unintentionally question the readers intelligence.

Methods

I am confused in the methods with the section called intact mass analysis. Intact mass analysis to me, is mass analysis of intact spike or RBD. Are the authors talking about glycan remaining on peptides after elastase digest of spike and RBD?

Conclusion

“pathogen such as SARS-CoV-2 whose ability to evade the immune system is dependent upon heavy and complex glycosylation.”
This has not been proven and is supasition - please temper with phrase such as “potentially”

“We believe the MRTF method described here has advantages over other approaches to Spike glycan analysis.” Delete we believe

Reviewer #2 comment	Authors' response
The paper described precisely the method, in particular, the authors made a few interesting remarks of the data in the discussion section about the appearance of the abundant peptides as well as the low abundant glycan. It would be more meaningful if the authors can relate the observations to the biology of SARS-COV-2.	We agree and have included our speculative thoughts on the biological importance of different Spike glycan profiles and how this could influence current research aimed at diagnostics and disease prevention.
On the other hand, because of the generality of the retention time-accurate mass database and RPLC gradient used in the paper, it would be better to see the authors cited a few articles about the realistic ideas of this type of database.	We agree that use of mass-retention time databases is commonplace and we have included references to this effect. Their use is less common for protein characterisation.
Scientifically, it is also better to mention the advantages of using this column compare to other known columns to convince other users for the investment.	We have added a description of the specific features of this column which lead us to select it. It may well be that another column may perform equal or better peptide separation. Evaluation of different columns was not feasible given the limited Spike protein available to us. Reproducibility of retention times is critical for the database to be of use, and duplicating the method using the same commercial quality controlled HPLC column we believe is the most reliable way to achieve this. Retention prediction is feasible if the peptide sequences are known, but as we explain, the non-specific elastase cleavage means that some species are listed in our database by mass and retention time only.
Moreover, for the data analysis, can the authors observe any trend of elutions based on different chemical differences of the glycopeptides? And if feasible, can the author present this relation using the linear regression to further making the in-silico prediction for the retention time feature of the library/database.	The reviewer has correctly identified a correlation between RT and differences in glycan structure and mass as shown below for the GVFNAT. This might be expected, but the correlation is relatively weak (Rsq=0.775), due to the lack of selectivity for sugars using reversed phase HPLC, and we do not think the separation is large enough to be of practical use.

	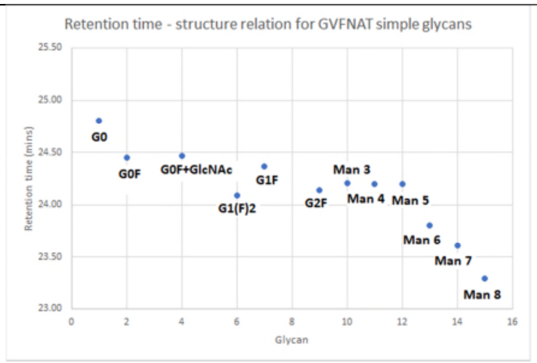
Moreover, there are some terms in the paper needed more clarifications or explanations in the results section to help to paper to reach a wide range of readers.	We revised the document to make the use of some terms consistent, so always 'Spike protein' instead of "Spike". We replaced PCDL with MFRT database and other changes to make it easier to read.
Reviewer # 3 comment	Authors' response
Remove "rapid and robust identification" there is insufficient evidence to substantiate this claim.	We agree.
Similarly no evidence that "any LC-ToF" can use this technique. Although it has huge potential these claims can only be made once the technique has been replicated by independent laboratories.	We agree.
Also define typical mass spectrometry laboratory, as clinical microbiology mass spectrometry laboratories do not use LC-MS but use exclusively MALDI-Tofs.	We have removed this phrase.
"We stress, however, that there is no " delete We stress as this is not needed starting "However" makes the point and does do unintentionally question the readers intelligence.	Deleted
I am confused in the methods with the section called intact mass analysis. Intact mass analysis to me, is mass analysis of intact spike or RBD. Are the authors talking about glycan remaining on *peptides after elastase digest of spike and RBD?*	We use "intact mass analysis" as a general term to describe top-down analysis of any protein (or part thereof such as RBD) as opposed to bottom-up methods dependent on proteolytic cleavage and analysis of the resulting peptides. The wording has been changed to clarify this point.
"We believe the MRTF method described here has advantages over other approaches to Spike glycan analysis." Delete we believe	Deleted.

REVIEWERS' COMMENTS:

Reviewer #2 (Remarks to the Author):

This revision of the paper took the previous suggestions into consideration and indeed helped the paper more adaptable and convincing. The data presented clearly and easy to follow. Great choice of title to deliver the whole content of the paper.

Reviewer #3 (Remarks to the Author):

The Authors have addressed fully all my comments. This is a sound and very valuable piece of work which will be a reference method that will yield untold future findings in glycobiology.
